# Chrysanthemum classification method integrating deep visual features from both the front and back sides

**DOI:** 10.3389/fpls.2024.1463113

**Published:** 2025-01-21

**Authors:** Yifan Chen, Xichen Yang, Hui Yan, Jia Liu, Jian Jiang, Zhongyuan Mao, Tianshu Wang

**Affiliations:** ^1^ School of Computer and Electronic Information/School of Artificial Intelligence, Nanjing Normal University, Nanjing, Jiangsu, China; ^2^ Nanjing University of Chinese Medicine, National and Local Collaborative Engineering Center of Chinese Medicinal Resources Industrialization and Formulae Innovative Medicine, Nanjing, China; ^3^ Jiangsu Collaborative Innovation Center of Chinese Medicinal Resources Industrialization, Nanjing University of Chinese Medicine, Nanjing, Jiangsu, China; ^4^ College of Artificial Intelligence and Information Technology, Nanjing University of Chinese Medicine, Nanjing, Jiangsu, China; ^5^ Jiangsu Province Engineering Research Center of Traditional Chinese Medicine (TCM) Intelligence Health Service, Nanjing University of Chinese Medicine, Nanjing, Jiangsu, China

**Keywords:** Chrysanthemum classification, two-stream network, visual information, feature fusion, deep learning

## Abstract

**Introducion:**

Chrysanthemum morifolium Ramat (hereinafter referred to as Chrysanthemum) is one of the most beloved and economically valuable Chinese herbal crops, which contains abundant medicinal ingredients and wide application prospects. Therefore, identifying the classification and origin of Chrysanthemum is important for producers, consumers, and market regulators. The existing Chrysanthemum classification methods mostly rely on visual subjective identification, are time-consuming, and always need high equipment costs.

**Methods:**

A novel method is proposed to accurately identify the Chrysanthemum classification in a swift, non-invasive, and non-contact way. The proposed method is based on the fusion of deep visual features of both the front and back sides. Firstly, the different Chrysanthemums images are collected and labeled with origins and classifications. Secondly, the background area with less available information is removed by image preprocessing. Thirdly, a two-stream feature extraction network is designed with two inputs which are the preprocessed front and back Chrysanthemum images. Meanwhile, the incorporation of single-stream residual connections and cross-stream residual connections is employed to extend the receptive field of the network and fully fusion the features from both the front and back sides.

**Results:**

Experimental results demonstrate that the proposed method achieves an accuracy of 93.8%, outperforming existing methods and exhibiting superior stability.

**Discussion:**

The proposed method provides an effective and dependable solution for identifying Chrysanthemum classification and origin while offering practical benefits for quality assurance in production, consumer markets, and regulatory processes. Code and data are available at https://github.com/dart-into/CCMIFB.

## 1 Introduction

Chrysanthemum morifolium Ramat (hereinafter referred to as Chrysanthemum), a traditional iconic flower in China, boasts a wide variety of species, versatile applications, and a long-standing cultivation history. Chrysanthemums exhibit remarkable aesthetic appeal and hold substantial economic as well as medicinal value. They abound in flavonoids (Sun et al., 2021), volatile oils (Zhan et al., 2021), chlorogenic acid (Chen et al., 2021a), sesquiterpenes (Jiang et al., 2021), triterpenes, and amino acids, delivering advantageous health attributes like anti-inflammatory (He et al., 2019), antimicrobial, antioxidant (Youssef et al., 2020), anti-HIV, and anticancer effects (Yang et al., 2017). Chrysanthemums have found wide applications in food, tea, ornamentation, and pharmaceuticals. Particularly, Chrysanthemum tea is highly cherished for its health benefits like heat-clearing, digestion-enhancing, liver-nourishing, and vision-boosting effects (Yuan et al., 2020). Nevertheless, their quality and pricing are contingent upon their place of origin. Identifying the classification and origin of Chrysanthemum rapidly and precisely is important for producers, consumers, and market regulators.

Traditional methods of origin identification mainly comprise plant phenotype analysis and physicochemical analysis. Plant phenotype analysis heavily relies on human intervention, involving expertly trained assessment teams to primarily discern the characteristics of Chrysanthemums, including shape, color, and odor. The huge group of China’s traditional Chrysanthemum categories, coupled with the yearly cultivation of numerous new breeds, contribute to an immensely diverse array of species. Additionally, Chrysanthemums display a rich array of colors and intricate floral structures. Its inflorescences are diverse in shape, with variable proportions of tubular and ligulate flower composition, especially characterized by an increase in the number of ligulate flowers. Many Chrysanthemum categories share a high degree of similarity, making manual identification methods time-consuming, prone to errors, and challenging for large-scale variety classification and identification tasks. In the realm of physicochemical analysis of plants, researchers have introduced various analytical instruments and conducted extensive studies. Techniques such as GC-MS, electronic nose (Luo et al., 2017), FT-IR (Liu et al., 2008), ICP-MS (Long et al., 2013), LC×LC-Q-TOF/MS (Chen et al., 2021b), in conjunction with chemometrics, have been utilized for the identification of Chrysanthemum origins. These methods primarily ascertain the geographical origin and category origin of Chrysanthemums by detecting the content of effective components, providing advantages of excellent repeatability and high sensitivity. Nevertheless, these analytical methods face challenges such as intricate sample preparation, prolonged analysis duration, and overreliance on costly equipment such as infrared spectrometers and electronic nose devices. Therefore, the urgency to develop a swift, non-invasive, and non-contact method for Chrysanthemum classification becomes especially pronounced.

In recent years, ample research has demonstrated that the future progress of plant phenotype analysis relies on the utilization of computer vision and deep learning methods. This method has surpassed the limitations of manually gathering plant phenotype data, which offers a robust avenue for plant phenomics research. Analyzing and processing plant image data facilitates the automatic measurement of plant morphological features, including leaf area, presenting a swift, efficient, and precise method for botanical morphological research. Some researchers have proposed a novel DFN-PSAN (Dai et al., 2024) model that effectively integrates multi-scale relevant features extracted from different network layers to accurately identify crop diseases in natural agricultural environments. Additionally, the ITF-WPI (Dai et al., 2023) model successfully identifies 17 common pests of goji berries by incorporating a context-aware Transformer network and a Pyramid Squeeze Attention (PSA) mechanism, achieving promising results. Some researchers have devised learning-enhanced methodologies integrating the Inception-v4 convolutional neural network for grading and fraud detection of saffron images taken with smartphones (Momeny et al., 2023). Additionally, Researchers utilize computer vision techniques to detect pests (Hadipour-Rokni et al., 2023), plant diseases (Momeny et al., 2022), maturity (Azadnia et al., 2023) in citrus fruits, and more. These endeavors illustrate the extensive prospects of computer vision technology in botanical morphological studies. Recent research has aimed to utilize image feature extraction methods for identifying and detecting Chrysanthemum categories. Some researchers undertook preliminary variety identification by extracting Gray Level Co-occurrence Matrix(GLCM) (Zhai et al., 2016) textures from images of 20 ornamental Chrysanthemum categories. Additionally, other researchers utilized hyperspectral imaging to measure spectral reflectance values from various parts of Chrysanthemum ray florets and analyzed their correlation with the measured pigment content. Moreover, researchers extracted Local Binary Pattern (LBP) (Liu et al., 2017) texture features from unfolded images of 24 Chrysanthemum categories. However, these methods were limited to recognizing the overall inflorescence shape and ray floret patterns of Chrysanthemums, relying only on single-side information and ignoring the potential of both front and back views. Therefore, they lacked the capability for precise identification of Chrysanthemum categories.

To address the aforementioned issues, this paper presents a Chrysanthemum classification via the fusion of deep visual features of both the front and back sides. This method employs a deep neural network to extract the color, texture, and shape features of Chrysanthemums, facilitating the classification of Chrysanthemum species. The key steps are as follows. Firstly, the different Chrysanthemum images are collected and labeled with origins and classifications. Secondly, the background area with less available information is removed by image preprocessing. Thirdly, a two-stream feature extraction network is designed with two inputs which are the preprocessed front and back Chrysanthemum images. Meanwhile, the incorporation of single-stream residual connections and cross-stream residual connections is employed to extend the receptive field of the network and fully fusion the features from both the front and back sides. The proposed method demonstrates a robust solution for chrysanthemum classification by integrating deep visual features from both front and back views. This method addresses the pressing need for rapid, non-invasive classification techniques in the agricultural and medicinal industries, enabling efficient quality assessment and traceability of Chrysanthemum products.

The primary contributions of this paper include:

1. Proposing subjective screening criteria for Chrysanthemum images, excluding non-compliant ones, thereby providing a clean and standardized dataset for classification.
2. Introducing a two-stream neural network model tailored to the unique structure and morphology of chrysanthemums, enabling extraction of critical features from both sides of the flower.
3. Presenting a strategy for integrating deep features from both sides through inter-layer and inter-path interactions, facilitating comprehensive fusion of front and back chrysanthemum features.

The subsequent sections of this paper are organized as follows: Section 2 delves into relevant work on image classification and Chrysanthemum species recognition. Section 3 elaborates on the structure and specifics of the proposed network. Section 4 delineates the results of performance evaluation experiments. Finally, Section 5 concludes the paper.

## 2 Related work

### 2.1 Image classification based on visual features

Image classification is a fundamental challenge in computer vision, aiming to categorize images into distinct classes. Traditional machine learning methods have been utilized for image classification over recent decades. Yet, with the evolution of deep learning, deep neural networks have emerged as the forefront method for image classification. ResNet (Residual Network), introduced by He et al. (He et al., 2016), enhances accuracy by considerably increasing depth. Its internal residual blocks use skip connections, alleviating the vanishing gradient issue in deep neural networks. Lin et al. (Lin et al., 2015). introduced BCNN (Bilinear CNN), which utilizes bilinear pooling to grasp pixel relationships among images for classification. It captures global feature information by computing the outer product of two CNN feature maps, making it a highly representative model for fine-grained weakly supervised learning. Researchers from Stanford University and Facebook AI Research introduced RegNet (Radosavovic et al., 2020) which is a novel convolutional neural network. It achieves heightened accuracy and reduced parameter count through automated network structure search. EfficientNet (Tan and Le, 2019), published by Google, improves model performance through balanced scaling in depth, width, and resolution. With the advent of attention mechanisms, numerous researchers have implemented them in image classification tasks. SENet (Hu et al., 2018) (Squeeze-and-Excitation Network), introduced by Hu et al., dynamically adjusts feature map weights using attention mechanisms to enhance image classification performance. CBAM (Woo et al., 2018) (Convolutional Block Attention Module), introduced by Woo et al., combines channel and spatial attention to optimize the model’s attention on various image regions, thereby improving image classification performance. Swin-T (Liu et al., 2021), proposed by researchers from The Chinese University of Hong Kong and Microsoft Research Asia, achieves increased accuracy and decreased computational complexity through multi-level partitioned attention mechanisms. TinyViT (Wu et al., 2022), introduced by researchers from Microsoft, is a novel compact ViT. It transfers knowledge from large pre-trained models to smaller ones using swift pre-training distillation methods, allowing smaller models to leverage abundant pre-training data. ConvNeXtV2 (Woo et al., 2023), an extension derived from the ConvNeXt (Liu et al., 2022) architecture inspired by MAE (He et al., 2022), introduces a new Global Response Normalization (GRN) layer to enhance competitive feature expression among channels within the original ConvNeXt modules, thereby better capturing discriminative channel features. Liu Liu et al. (2023) et al. propose EfficientViT, a family of high-speed vision transformers that enhance memory efficiency and reduce computational redundancy, achieving high accuracy. Wang Wang et al. (2024) et al. re-examined the efficient design of lightweight CNNs and highlighted their potential on mobile devices. By integrating the efficient architecture design of lightweight ViTs, they progressively enhanced standard lightweight CNNs, resulting in RepViT, which demonstrates excellent performance-latency balance.

Presently, within the realm of Chrysanthemum research, deep learning methods are progressively emerging. Fu et al. (Long et al., 2023). combined hyperspectral imaging technology with chemometrics to explore and apply the discrimination of Hangbaiju’s origins. Yuan et al (Yuan et al., 2018). employed convolutional neural networks for Chrysanthemum flower identification, achieving an identification rate of approximately 95%. Nonetheless, it categorized only 5 flower types and could not accurately discern categories. Following that, they introduced a Chrysanthemum image phenotype classification framework based on transfer learning and bilinear convolutional neural networks (Yuan et al., 2022). Utilizing a symmetrical VGG16 network as a feature extractor, they eventually fed global features into the classification layer for sorting. Liu et al. (Liu et al., 2019b). captured 14,000 images of 103 large Chrysanthemum categories using an image acquisition device. Leveraging the concept of transfer learning, they established a recognition model for these categories based on VGG16, GoogLeNet, and ResNet50 deep convolutional neural networks. However, the clustering and visualization of extracted deep features did not manifest distinct distribution patterns. Wang et al. (Wang et al., 2022). integrated AP clustering analysis with deep features, proposing a multi-information model based on deep learning for the identification and classification of large-flowered Chrysanthemums. Building upon the traditional VGG16 convolutional neural network, Huang et al. (Huang and Liu, 2023). introduced an enhanced multi-scale, multi-parallel convolutional neural network termed VGG-Inception. Utilizing parallel network structures and global pooling layers maintains model depth and augments network width while reducing parameters to just 9% of the original VGG16. The utilization of auxiliary classifiers mitigates gradient vanishing, thereby enhancing the model’s ability to generalize.

### 2.2 Deep Multi-Path Networks

Deep Multi-Path Networks represent intricate neural network structures employing multiple parallel processing pathways to concurrently handle diverse inputs or feature channels, striving to comprehensively capture image information and bolster processing efficiency. This architecture permits diverse pathways to specialize in distinct feature extraction or tasks. By amalgamating the outputs from these pathways, it generates more precise or comprehensive outcomes, exhibiting significant relevance across multiple domains of image processing. Ma et al. (Ma and Oh, 2022). introduced a two-stream network based on wavelet transform to address color deviations and blurry details in underwater images. Pan et al. (Pan et al., 2021). introduced a method to enhance synthesized view quality based on a two-stream attention network (TSAN). The global information extraction stream learns contextual information, while the local information extraction stream extracts texture details from rendered images. The Multi-Scale Residual Attention Block (MSRAB) effectively detects features of varying scales and optimizes them by considering spatial interdependencies. Liu et al. (Liu et al., 2019a). proposed a driver fatigue detection method utilizing a two-stream network model integrating various facial features. It extracts static features from partial facial images and dynamic features from partial facial optical flow, feeding them into a two-stream neural network for feature fusion and classification, showcasing excellent performance. Zhou et al. (Zhou et al., 2023). harnessed EfficientNet’s efficient feature extraction abilities to separately extract spatial and temporal features of consecutive video frames from spatial and temporal flows. They then employed a multi-head attention mechanism to capture pivotal action details, facilitating action recognition using the amalgamated features. Wang et al. (Wang et al., 2020). introduced a global-local two-stream architecture for multi-scale representation, aimed at resolving classification performance constraints due to extensive feature variations in remote sensing images. The Correlation-Driven Joint Bone-Flow Graph Convolutional Network (CD-JBF-GCN), devised by Tu et al. (Tu et al., 2022), delves into the motion transmission amid joint and bone flows, facilitating more discernible feature representations in both streams. This network demonstrates cutting-edge performance in semi-supervised skeleton action recognition. Zheng et al. (Zheng et al., 2023). introduce a novel Cross-Attention and Cross-Scale Fusion Network (CASF-Net) that maximizes the potential of two-stream networks and fully integrates coarse-grained and fine-grained feature representations. The designed dual-branch encoder focuses on modeling non-local dependencies and multi-scale contexts, markedly improving semantic segmentation quality. Similarly, Xie et al. (Xie et al., 2023). introduce the Context-Aware Network with Two-Stream Pyramid (CANet) tailored for medical image segmentation. Through multiple resolution input versions and multi-scale convolutional units, CANet adeptly captures diverse hierarchical multi-scale complementary features in medical images. SETNet, presented by Ma et al. (Ma et al., 2022), stands as a two-stream convolutional network for no-reference image quality assessment. While the image stream attends to the entire image content, the saliency stream explicitly guides the network in learning spatially significant features more appealing to human perception. Leveraging spatial and channel attention modules refines features and amalgamates multi-level features to predict image quality scores. Drawing inspiration from the human visual system, Chen et al. (Chen et al., 2023). present a two-stream convolutional neural network for blind image quality assessment tasks. Emulating the two pathways of the human eye, the model extracts image content and global shape features, integrating multi-scale features to enhance assessment performance.

## 3 Method

**Figure 1:**
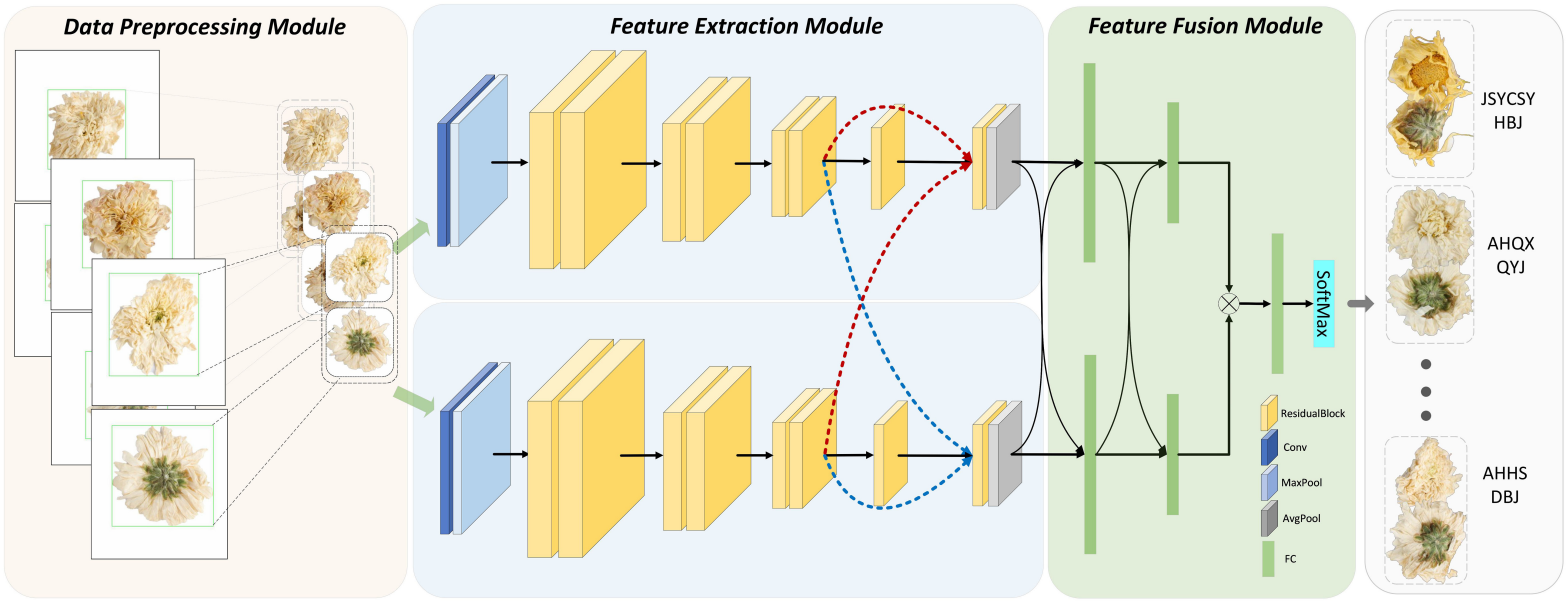
The architecture of the proposed method with data preprocessing module, two-stream feature extraction module, and feature fusion module.

To achieve efficient identification of chrysanthemum species, this paper outlines the specific workflow depicted in **Figure 1**, where the different parts have been clearly labeled. It primarily encompasses three components: 1) data preprocessing, 2) feature extraction, and 3) feature fusion.

### 3.1 Data preprocessing

Before network input, all Chrysanthemum images undergo a series of preprocessing steps. Firstly, the Canny edge detection method is applied to detect the edges of the Chrysanthemum flowers, making the boundaries clearer. Secondly, a minimal square encompassing all edges is used to crop the image, removing any irrelevant background. By doing so, this step focuses the analysis on the flower, eliminating distractions that could reduce model accuracy. Thirdly, all images are resized to 224x224 pixels to ensure consistent input dimensions, avoiding accuracy reduction caused by varying image scales. Fourthly, to further diversify the training set and reduce overfitting, random rotations of up to 15° are applied. This augmentation step simulates real-world scenarios, where flowers may appear at different angles, enhancing the model’s robustness. Fifthly, image normalization is performed to standardize pixel values across the dataset, aiding in faster model convergence and improving generalization. This normalization reduces the risk of gradient instability during training by scaling pixel values to a common range. **Figure 1** illustrates the preprocessing pipeline, highlighting edge detection (green box), cropping, and resizing for network input.

### 3.2 Feature extraction

Acknowledging the distinct features between the front and back of various Chrysanthemum species, the method proposed in this study employs a two-stream neural network model, utilizing an enhanced ResNet-18 model for both streams. Inputs consist of images capturing the front and back of Chrysanthemums, tailored to extract crucial features specific to each side, considering the unique structure and morphology of the flowers. A traditional ResNet-18 structure comprises 17 convolutional layers, 1 Maxpooling layer, 1 Avgpooling layer, 1 fully connected layer, and 1 Softmax layer. In contrast to this, our proposed network extends the end fully connected layer to three. The network architecture primarily consists of shallow feature extraction, encompassing the initial 13 convolutional layers, and deep feature fusion, comprising the final 4 convolutional layers and 2 fully connected layers. In the end, the upper and lower stream features are concatenated and merged through a single fully connected layer, as depicted in **Table 1** outlining the network structure parameters.

**Table 1:** The parameters of the network.

Block name	Layer name	parameters	Layer name	parameters
Conv1	Conv	7×7, 64	Conv	7×7, 64
	Maxpool	3×3	Maxpool	3×3
Conv2	Conv	$\left[\begin{array}{c} 3\times 3,\;64\\ 3\times 3,\;64 \end{array}\right]\times 2$	Conv	$\left[\begin{array}{c} 3\times 3,\;64\\ 3\times 3,\;64 \end{array}\right]\times 2$
	Bn+relu		Bn+relu	
Conv3	Conv	$\left[\begin{array}{c} 3\times 3,\;128\\ 3\times 3,\;128 \end{array}\right]\times 2$	Conv	$\left[\begin{array}{c} 3\times 3,\;128\\ 3\times 3,\;128 \end{array}\right]\times 2$
	Bn+relu		Bn+relu	
Conv4	Conv	$\left[\begin{array}{c} 3\times 3,\;256\\ 3\times 3,\;256 \end{array}\right]\times 2$	Conv	$\left[\begin{array}{c} 3\times 3,\;256\\ 3\times 3,\;256 \end{array}\right]\times 2$
	Bn+relu		Bn+relu	
Conv5	Conv	$\left[\begin{array}{c} 3\times 3,\;512\\ 3\times 3,\;512 \end{array}\right]\times 2$	Conv	$\left[\begin{array}{c} 3\times 3,\;512\\ 3\times 3,\;512 \end{array}\right]\times 2$
	Bn+relu		Bn+relu	
	Avgpool	7×7	Avgpool	7×7
FC Block	FC1: output nodes128		FC2: output nodes128	
	FC3: output nodes32		FC4: output nodes32	
	FC5: output nodes18			

Within **Table 1**, “Block name” represents module names, while “Layer name” denotes operation names—’conv’ for convolution, ‘BN’ for batch normalization, ‘relu’ for activation function, and ‘Maxpool’ for max-pooling operation. ‘Parameters’ refer to the convolutional kernel size and output channel quantity. Starting with 224*224*3 chrysanthemum phenotype image data, a 7*7 convolutional layer with a stride of 2 and padding of 3 produces output data sized 112*112*64. Subsequent max-pooling, using a 3*3 kernel, a stride of 2, and padding of 1, results in data sized 56*56*64, halving the feature’s dimensions without altering the channel count. Following this, Conv2, with a stride of 2 and padding of 1, maintains the data’s size and channel count. Then, Conv3, Conv4, and Conv5 double the channel count while halving the output data size, resulting in 7*7*512 output dimensions. A final global average pooling layer outputs data sized 1*1*512. The first two fully connected layers transform data to 128 dimensions and 32 dimensions, respectively. These two 32-dimensional vectors concatenate into a 64-dimensional vector, fed into the last fully connected layer, yielding the ultimate feature vector.

### 3.3 Feature fusion

Inspired by residual networks, this paper introduces cross-stream residual connections to simultaneously extract features and enhance the network’s field of view, allowing better integration of both positive and negative chrysanthemum features.

**Figure 2:**
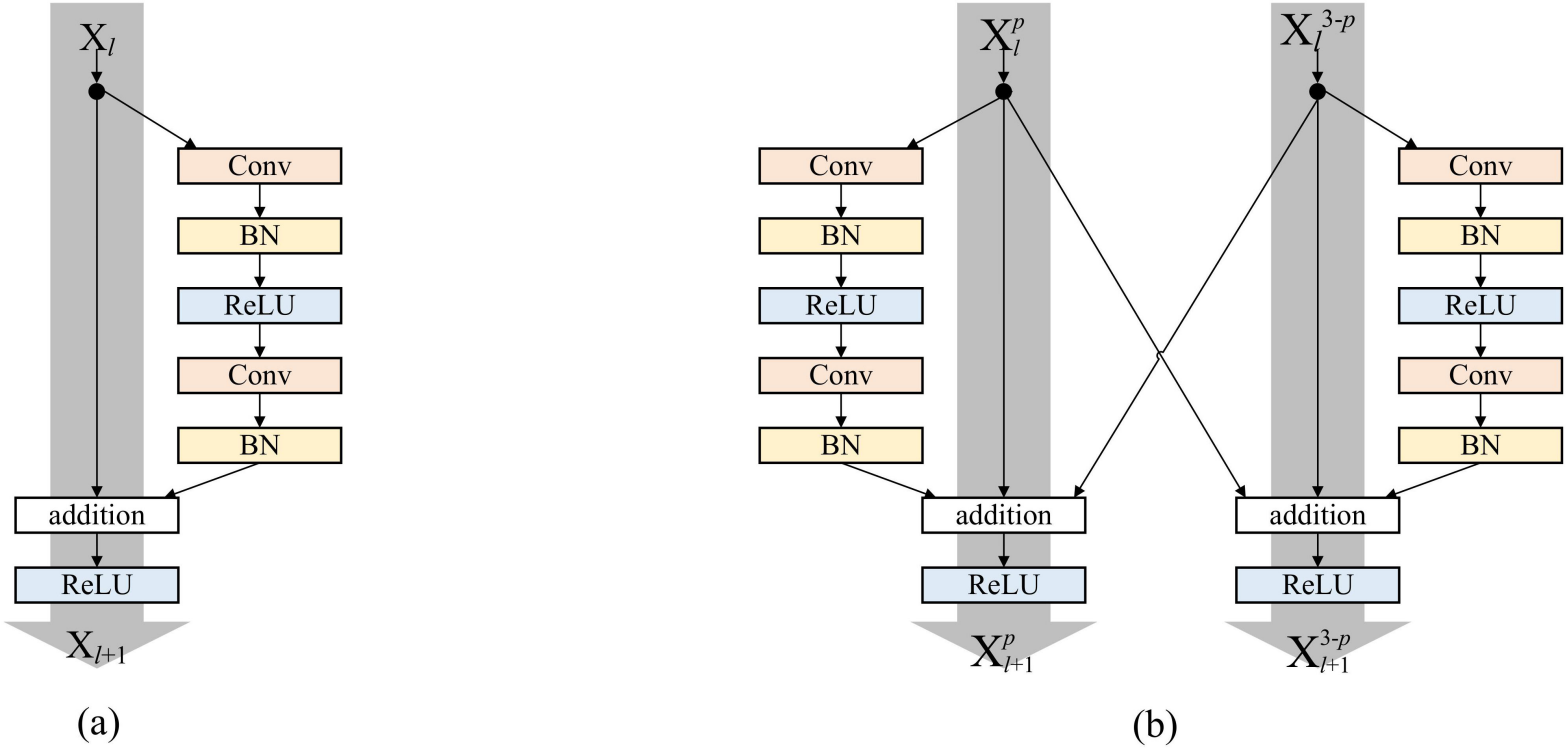
**(A)** The traditional residual connections. **(B)** The cross-stream residual connections proposed in this paper.


As illustrated in **Figure 2A**, the interaction mode of residual connections between front and back layers is a traditional single-stream ResNet network. Here, $x_{l}$ represents the output of the previous layer, $h\left(x_{l}\right)$ denotes direct mapping [the left line in (a)], while $F\left(x_{l},W_{l}\right)$ signifies residual [the convolutional part on the right side in (a)], where *l* indicates the layer and $W_{l}$ represents the *l* − *th* convolutional layer. The formula for the output of this layer $x_{l+1}$ is as follows.

(1) $$x_{l+1}=h\left(x_{l}\right)+F\left(x_{l},W_{l}\right)$$

**Figure 2B** illustrates the interaction mode of residual connections between the upper and lower paths within the two-stream convolutional neural network proposed in this paper. Here, $x_{l}^{p}(p\;\subset \;1,2)$ signifies the output of the upper or lower path network in the previous layer. When *p* equals 1, $x_{l}^{p}$ represents the output of the upper path network processing the front image in the prior layer, and when *p* equals 2, $x_{l}^{p}$ corresponds to the output of the lower path network processing the back image in the previous layer. *h* denotes an identity mapping, *Re* indicates the joint residual of the upper and lower paths in the prior layer. This layer’s output is denoted as $x_{l+1}^{p}$ represents the convolutional layer’s transformation, and the respective formula is delineated below:


(2) $$Re=h\left(x_{l}^{p}\right)+h\left(x_{l}^{3-p}\right)$$

(3) $$x_{l+1}=Re+F\left(x_{l}^{p},W_{l}^{p}\right)$$

During residual connections between preceding and subsequent layers, a cross-stream residual connection was applied, summing up the three outputs. The use of single-stream and cross-stream residual connections expands the network’s field of view, aiding in capturing diverse scale and abstract features within images. It facilitates direct information exchange between layers within the same path and between different paths, enabling deeper layers to benefit from shallower layers, and ultimately allowing a comprehensive fusion of features from both the front and back sides.

Similar strategies are employed in the fully connected layers to further amalgamate features from both the front and back sides:


(4) $$y_{l+1}^{p}=G\left(y_{l}^{p}+\frac{y_{l}^{3-p}}{3}\right)$$

$y_{l}^{p}(p\;\subset \;1,2)$ signifies the output of the upper or lower path network in the preceding layer. For *p* equal to 1, $y_{l}^{p}$ represents the upper path network’s output, and for *p* equal to 2, it corresponds to the lower path network’s output. Meanwhile, $y_{l}^{p+1}$ denotes the network’s output at this layer, with *G* representing the transformation in the fully connected layer. Unlike the convolutional layers, this process involves a weighted sum of the upper and lower path features, with a ratio of 1:3. This weighting ratio’s validation in subsequent experimental segments underscores its crucial role in the model’s performance.

This approach accounts for the potential similarity in front features but dissimilarity in back features among different Chrysanthemum species, or vice versa. This enhances the comprehensive integration of both front and back features, leading to more accurate identification of Chrysanthemum types.

### 3.4 Model training

In the training section of the network model, the dataset was divided into training, validation, and test sets at a ratio of 6:2:2. Staged training methods were utilized to enhance efficacy. Pretraining parameters from the ImageNet dataset were initially transferred, serving as the initial parameters for the first 13 layers of the two-stream neural network to expedite convergence and enhance effectiveness. These parameters were then frozen for preliminary training, facilitating the rapid acquisition of general features by the model. This phase comprised 20 iterations, a learning rate of 0.001, and a batch size of 24. Subsequently, the parameters for shallow feature extraction were unfrozen for model fine-tuning, involving 30 iterations, a learning rate of 0.0001, and a batch size of 24. The Adam optimizer was used to optimize the model, employing cross-entropy loss as the selected loss function. After each iteration, validation was conducted on the validation set, saving models that showed superior results to achieve the network’s optimal solution. To mitigate experimental errors caused by random sampling, the train-test prediction process was repeated 50 times, and the average accuracy was computed.

## 4 Experimental results

### 4.1 Database and evaluation metrics

**Figure 3:**
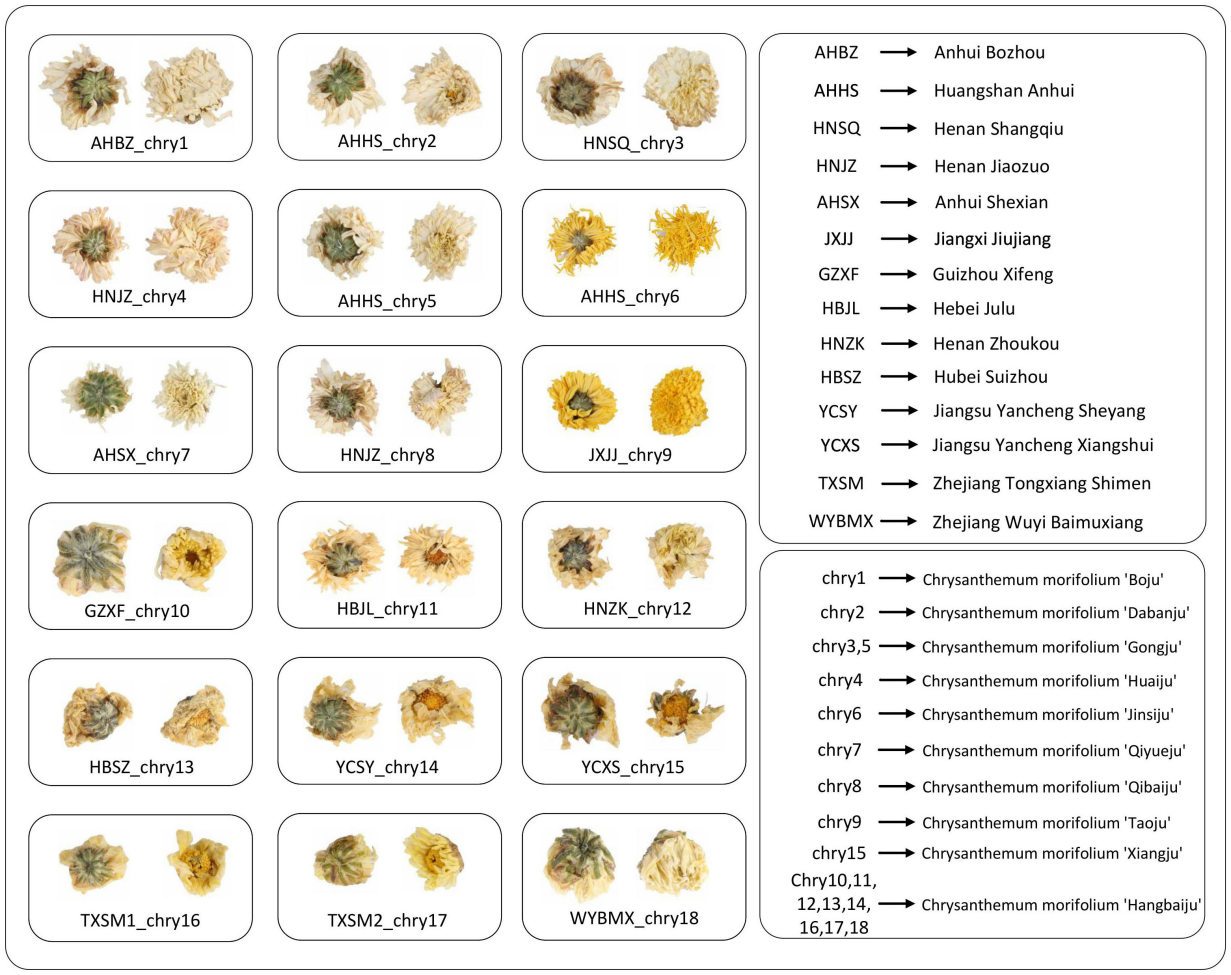
The illustration of Chrysanthemums from different origins and classifications. The pure letter abbreviations represent the origin, and the “chry” + number abbreviations represent the classification.

The chrysanthemums used in this study were collected between October 2022 and December 2022 from Bozhou Anhui, Jiaozuo Henan, Xifeng Guizhou, Tongxiang Zhejiang, and Julu Hebei, etc. The chrysanthemums were divided into 18 groups via the origin and species. There are three batches in each group, and each batch weighs about 1 kg. The chrysanthemums were identified as the inflorescence of Chrysanthemum morifolium Ramat. by Prof. Yan Hui, Nanjing University of Chinese Medicine. The images of the chrysanthemum were taken using a Canon EOS 5DS R camera in a fixed position under consistent natural lighting. Samples of Chrysanthemum morifolium ‘Hangbaiju’ were collected from Zhoukou, Henan; Sheyang, Yancheng, Jiangsu; Xifeng, Guizhou; Shimen and Wuyi Baimuxiang, Tongxiang, Zhejiang; Julu Hebei and Suizhou, Hubei. Samples of Chrysanthemum morifolium ‘Gongju’ were obtained from Huangshan, Anhui, and Shangqiu, Henan. Other categories included Chrysanthemum morifolium ‘Boju’ from Bozhou, Anhui, Chrysanthemum morifolium ‘Qiyueju’ from Shexian, Anhui, Chrysanthemum morifolium ‘Dabanju’ and Chrysanthemum morifolium ‘Jinsiju’ from Huangshan, Anhui, Chrysanthemum morifolium ‘Taoju’ from Jiujiang, Jiangxi, Chrysanthemum morifolium ‘Xiangju’ from Xiangshui, Yancheng, Jiangsu, and Chrysanthemum morifolium ‘Huaiju’ and Chrysanthemum morifolium ‘Qibaiju’ from Jiaozuo, Henan. The dataset comprises 18 Chrysanthemum species, with approximately 100 images of each Chrysanthemum’s front and back sides, resulting in a total of around 3600 images. **Figure 3** illustrates schematic images of chrysanthemums from different origins.

During image collection, four criteria were applied to select images that met the standards: While capturing images of the front and back of Chrysanthemums, attention was paid to angle selection. Front images were ensured to exclude receptacles, while the focal point of back images was positioned between the receptacle and calyx, typically with a larger receptacle portion. It is crucial to maintain the integrity of the Chrysanthemum’s receptacle and petals, as the absence of the receptacle may lead to irregular shapes, and missing petals may result in occlusion issues. The color of the flowers should resemble the majority within their respective categories, avoiding excessively dark or bright shades. Additionally, the size of the flowers should match the majority of flowers in their respective categories, avoiding situations where they are too large or too small.

This study employs six metrics—Accuracy, Recall, F1 Score, number of parameters (Param), Average Inference Time (AIT), and Standard Deviation (STD) between categories—to assess the performance of Chrysanthemum image classification methods. Accuracy indicates the proportion of correctly classified samples by the classifier, while Recall measures the classifier’s recognition capability for each category. The F1 Score, a combination of Precision and Recall, offers an overall evaluation of the classifier’s performance. Param reflects the complexity of the model by quantifying the number of trainable parameters. AIT measures the time taken for the model to process a single sample, providing insight into the model’s efficiency. The Standard Deviation between categories reflects the method’s diversity and balance across various Chrysanthemum categories. These metrics provide multiple perspectives that facilitate a comprehensive understanding of the classifier’s performance in Chrysanthemum classification tasks.

### 4.2 Effectiveness of backbone and optimizer selection

This study conducted a series of comparative experiments to validate the effectiveness of employing the ResNet network as the backbone for Chrysanthemum classification. CNN, Alex, VGG, GoogLeNet, and DenseNet were separately chosen as the backbone for the two-stream neural network. The detailed experimental results are provided in **Table 2**.

**Table 2:** Performance comparisons of different networks as backbone.

Backbone	Acc (%)	Recall (%)	F1 (%)	STD
TwoStreamAlex	89.1	88.0	88.1	7.22
TwoStreamVGG	84.9	83.4	83.7	9.44
TwoStreamGoogLeNet	90.9	89.9	90.0	7.58
TwoStreamCNN	88.6	87.9	88.0	7.24
TwoStreamDenseNet	93.0	92.1	92.2	5.93
Ours	93.8	93.5	93.4	5.20

The data from **Table 2** indicates that using ResNet as the backbone yielded the best performance across all evaluation metrics. Our model (ours) achieved the highest levels of accuracy, recall, and F1 score, reaching 93.8%, 93.5%, and 93.4% respectively. These rankings place our model at the forefront among all models, highlighting its superior classification performance. Our model showed a class-wise standard deviation of 5.20%, 0.7% lower than the second lowest, the two-stream DenseNet. This indicates the highest stability of our model’s performance among different categories. These findings further affirm the effectiveness of our model and its significant advantage. Thus, these experimental results showcase the superiority and rationale of using ResNet as the backbone.

**Figure 4:**
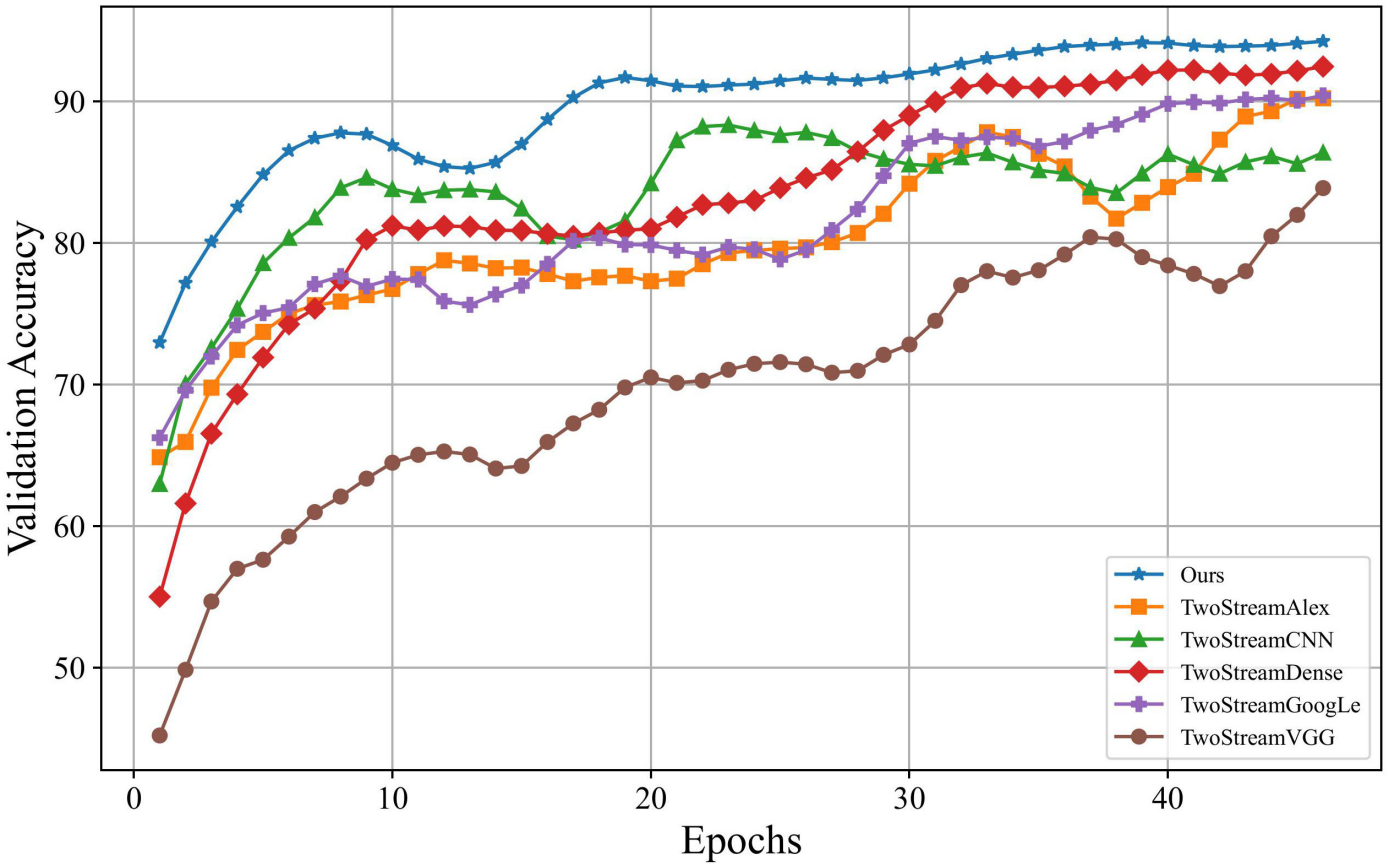
Convergence speeds of different networks as backbone.

On the other hand, this paper compared the iteration processes of training models using different networks as backbones. The specific results are depicted in **Figure 4**, where the horizontal axis represents the number of iterations, and the vertical axis represents accuracy on the validation set. Based on the outcomes in **Figure 4**, our model outperformed models using other networks as backbones. Although the two-stream DenseNet showed a result similar to the proposed model, its convergence speed was notably slower, which requires approximately 30 iterations to catch up with the proposed model. Notably, our model demonstrated rapid convergence in the early stages of training, which achieves high accuracy with only a few training iterations and maintains a lead. These outcomes underscore the superiority of using ResNet as the backbone concerning training efficiency.

**Figure 5:**
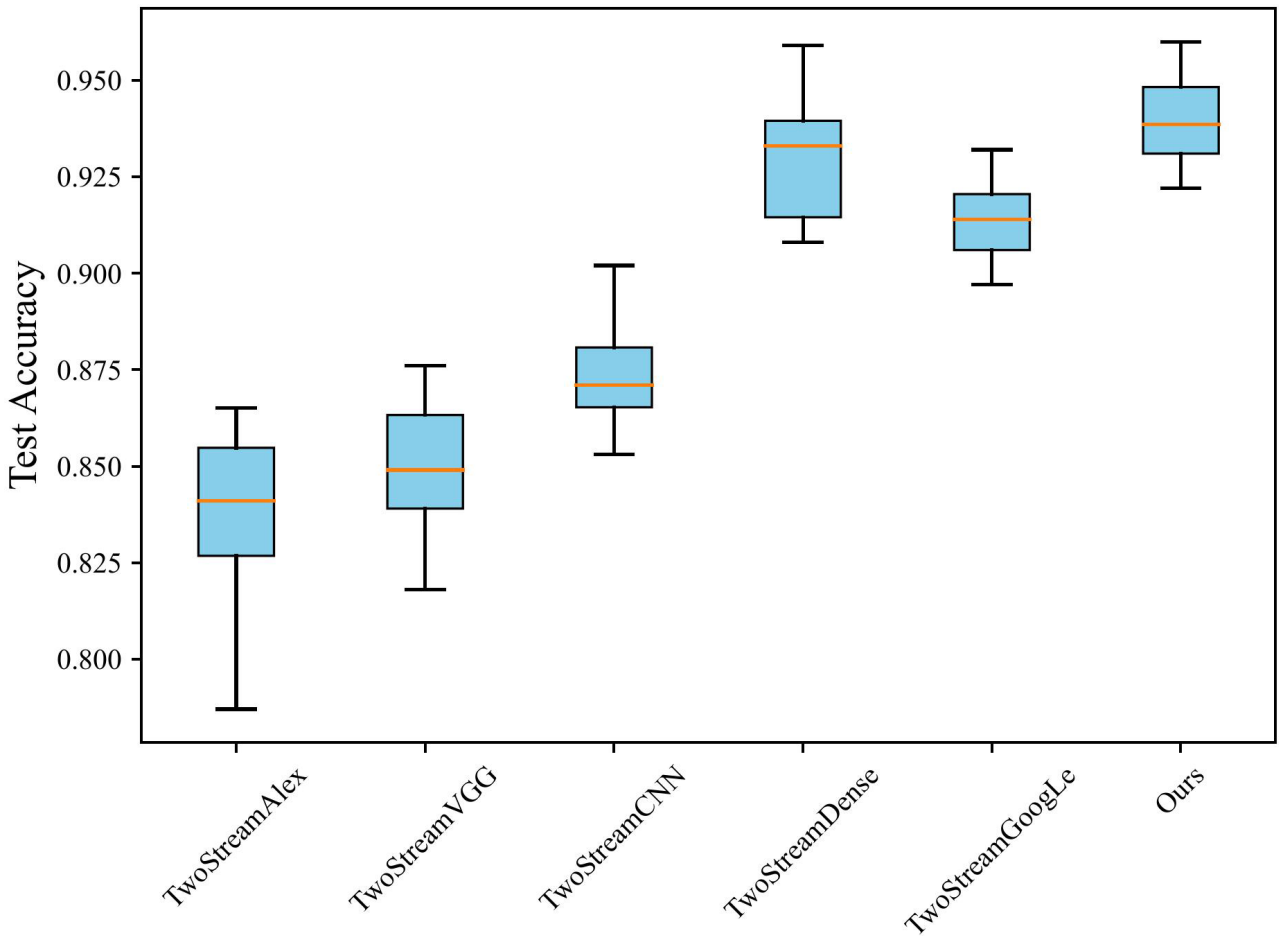
The accuracy stability of different networks as backbone.

Concurrently, **Figure 5** demonstrates a stability test of different networks used as backbones. The boxplot’s horizontal axis represents various image classification methods, and the vertical axis represents the accuracy metrics corresponding to each method. The box shape illustrates the data distribution of the corresponding results. While using DenseNet as the backbone achieves the second-highest performance, there is still a noticeable gap compared to the proposed method. In contrast, GoogleNet demonstrates high stability, but its performance notably lags behind the proposed method. Overall, employing ResNet as the backbone maintains high stability while achieving high accuracy. The comparisons in performance, convergence speed, and stability indicate that using ResNet as the backbone allows for faster model training while achieving optimal performance and stability.

**Figure 6:**
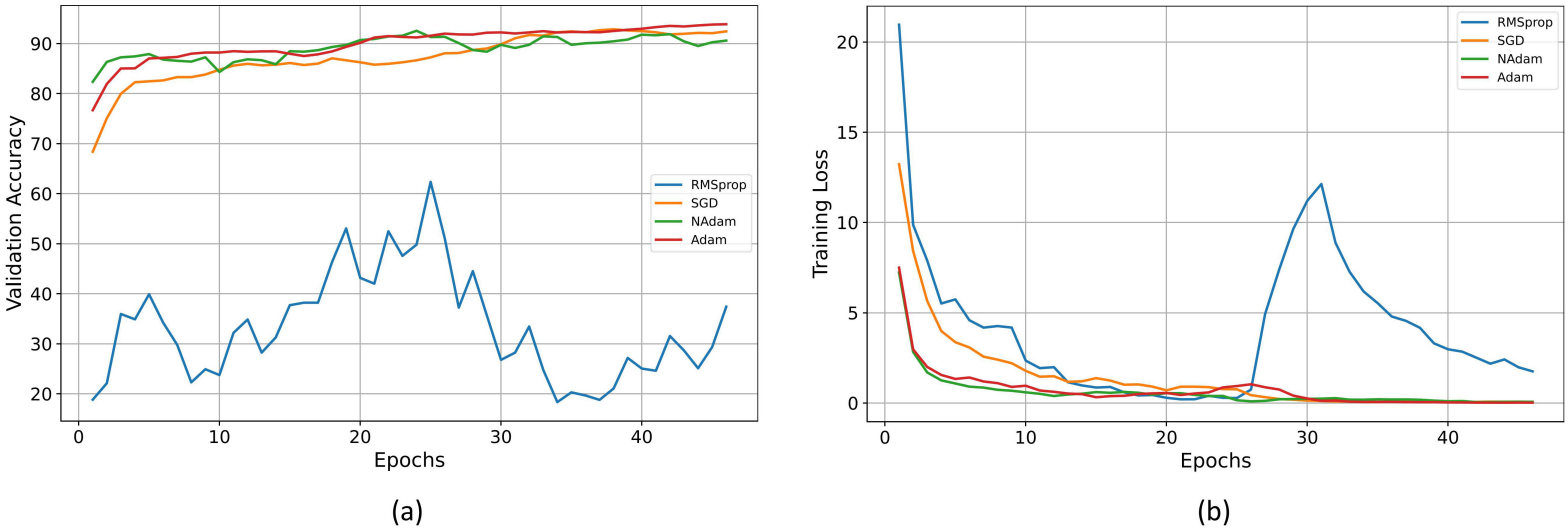
**(A)** Validation accuracy of different optimizers. **(B)** Training loss of different optimizers.

This paper designed a series of experiments to explore the impact of different optimizers on the model training process. The learning rate for all optimizers was uniformly set to 0.001, and when using the SGD optimizer, the momentum parameter was set to 0.9. The experimental results are shown in **Figure 6**: Figure A illustrates the accuracy curves of each optimizer on the validation set, while Figure B shows the trend of loss reduction during the training process. As observed from the figures, the RMSprop optimizer performed poorly in this task, with significant oscillations during training and difficulty in convergence. Although both SGD and NAdam achieved final losses close to zero, SGD exhibited a significantly slower convergence rate compared to Adam, while NAdam showed slight fluctuations in accuracy. The final accuracy of both optimizers was lower than that of Adam. The experimental results clearly demonstrate the effectiveness of the Adam optimizer for this task.

### 4.3 Performance comparison

In the comparison experiment, each type of Chrysanthemum type dataset is divided into a training set, a validation set and a test set according to the ratio of 6:2:2. To avoid experimental errors due to random sampling, the train-test process was repeated 50 times. The model with the lowest error on the validation set was selected as the final model, and the average values of Acc, Recall, F1, and STD over 50 experiments were calculated and considered as the final results. To demonstrate the effectiveness of the proposed method, this paper compares it against seven image classification methods, including four convolutional neural network approaches: BCNN, EfficientNetV2, RegNet, and ConvNeXtV2, as well as three ViT-based methods: TinyVit, EfficientViT, and RepViT. Considering the smallest version of ResNet was utilized as the backbone, the corresponding smallest versions of the other models were chosen for comparison, such as EfficientNetV2-s, ConvNeXtV2-atto, etc. Additionally, all models utilized pre-trained parameters from ImageNet to ensure a fair comparison in terms of resource consumption and model complexity.

According to **Table 3**, the proposed method exhibits competitive performance in accuracy, achieving 93.8%, slightly higher than ConvNeXtV2 and TinyVit’s 93.5%, and other classical models such as EfficientNetV2 with 91.7% and RegNet with 92.1%. Moreover, the proposed model displays excellent performance in terms of standard deviation between categories, recording only 5.20, considerably lower compared to other models ranging from 5.61 to 8.52. Through the analysis of experimental data, we observed that while EfficientViT has fewer parameters than our proposed model, it significantly lags in other performance metrics, including accuracy and AIT. On the other hand, RepViT has a similar number of parameters to our model and comparable accuracy, but its AIT is substantially slower than that of our proposed model. This not only showcases exceptional overall performance but also highlights the model’s stability and balance. These results underscore the effectiveness and robustness of the proposed model, validating its superiority in Chrysanthemum image classification tasks.

**Table 3:** Performance comparisons of different image classification methods.

Method	Acc (%)	Recall (%)	F1 (%)	STD	Param (M)	AIT (ms)
BCNN (2017)	88.1	88.2	87.4	8.52	15.9	0.26
RegNet (2020)	92.1	91.7	91.7	6.05	4.8	0.81
EfficientNetV2 (2021)	91.7	91.4	91.3	6.44	20.2	1.45
ConvNeXtV2 (2023)	93.5	92.9	92.9	6.32	3.4	0.61
TinyVit (2022)	93.5	93.2	93.1	5.61	11.0	0.69
EfficientViT (2023)	90.8	90.3	90.3	7.67	9.7	1.21
RepViT (2024)	92.9	92.5	92.5	6.11	12.1	1.19
Ours	93.8	93.5	93.4	5.20	13.4	0.33

When considering Param and AIT, the proposed method strikes an excellent balance between complexity and efficiency. With 13.4M parameters, our model is more lightweight compared to EfficientNetV2’s 20.2M and BCNN’s 15.9M. Although our model is slightly inferior to other methods, it still maintains competitive accuracy. Additionally, the model’s average inference time is 0.33ms, faster than ConvNeXtV2 (0.61ms), EfficientViT (1.21ms), and RepViT (1.19ms), demonstrating its computational efficiency. These results underscore the effectiveness of the proposed model, validating its superiority in Chrysanthemum image classification tasks.

**Figure 7:**
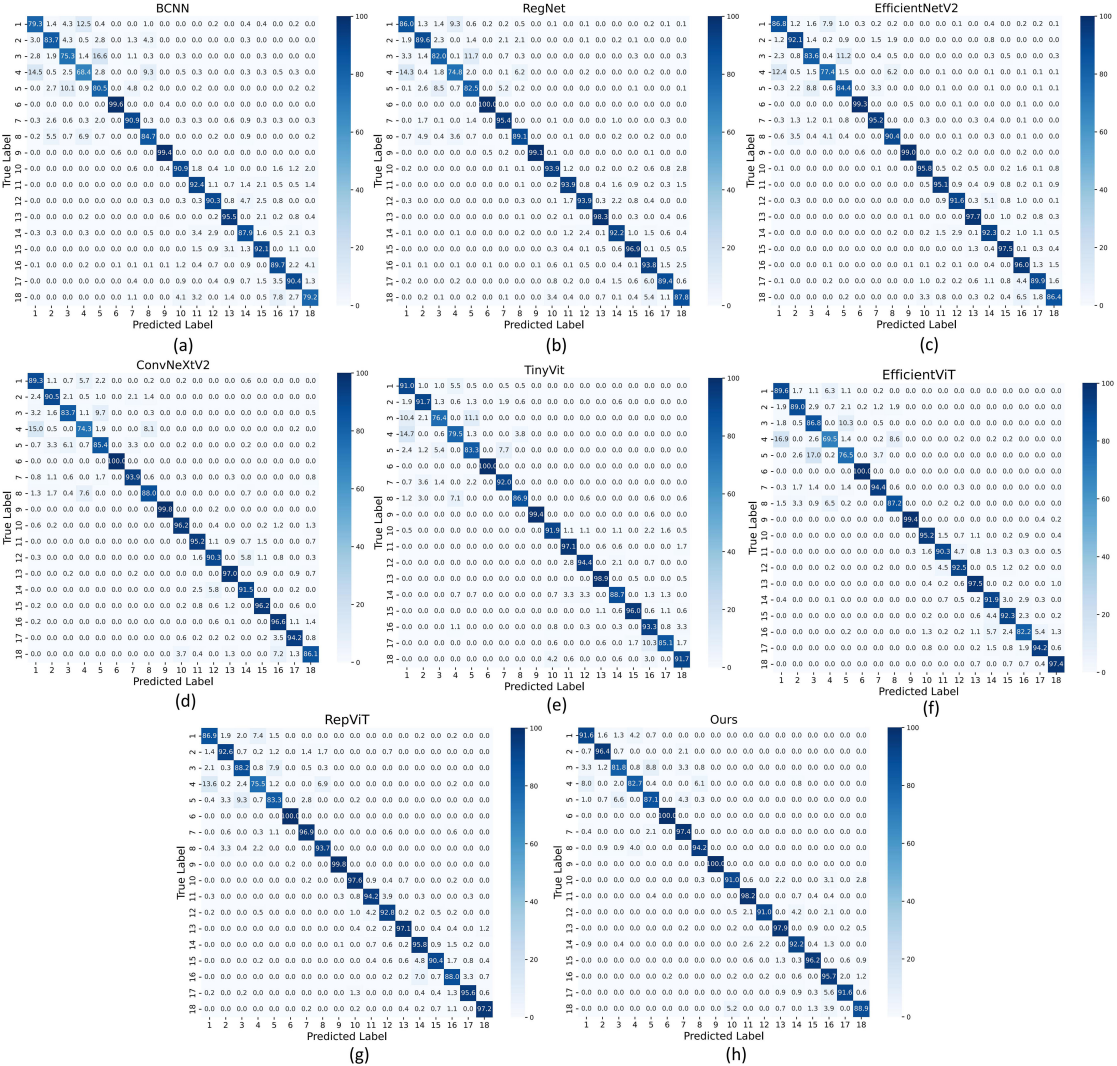
**(A)** Confusion matrix of BCNN. **(B)** Confusion matrix of RegNet. **(C)** Confusion matrix of EfficientNetV2. **(D)** Confusion matrix of ConvNeXtV2. **(E)** Confusion matrix of TinyVit. **(F)** Confusion matrix of EfficientViT. **(G)** Confusion matrix of RepViT. **(H)** Confusion matrix of Ours.

Additionally, to visually present the accuracy of different methods on each category, we plotted a confusion matrix as shown in **Figure 7**. From the figure, we can observe that our proposed algorithm achieves accuracy above 80% in each category, with only 4 categories having accuracy below 90%. In contrast, ConvNeXtV2 shows accuracy below 80% for AHHS-chry4 and below 90% for 6 categories, while TinyVit also has accuracy below 80% in two categories. Similarly, EfficientNetV2 achieves its highest accuracy only on YCSY-chry14 and YCXY-chry15, whereas our model achieves the highest accuracy in 9 categories. Further analysis of the confusion matrix reveals that other methods encounter difficulties with chrysanthemums such as chry1, chry3, chry4, and chry5, which have similar morphologies, leading to confusion and misclassification. For instance, EfficientNetV2, ConvNeXtV2, RepViT, EfficientViT and TinyVit misclassify 12.4%, 15%, 13.6%, 16.9% and 14.7% of chry4 as chry1, respectively, whereas our proposed model misclassifies only 8.0%. Similarly, these models often misclassify chry3 as chry5, whereas our model maintains a relatively lower error rate. This indicates that ConvNeXtV2, EfficientNetV2, RepViT, EfficientViT, and TinyVit perform relatively weaker on certain categories of chrysanthemum images, while our proposed algorithm demonstrates better overall classification ability and more robust performance in specific categories.

**Figure 8:**
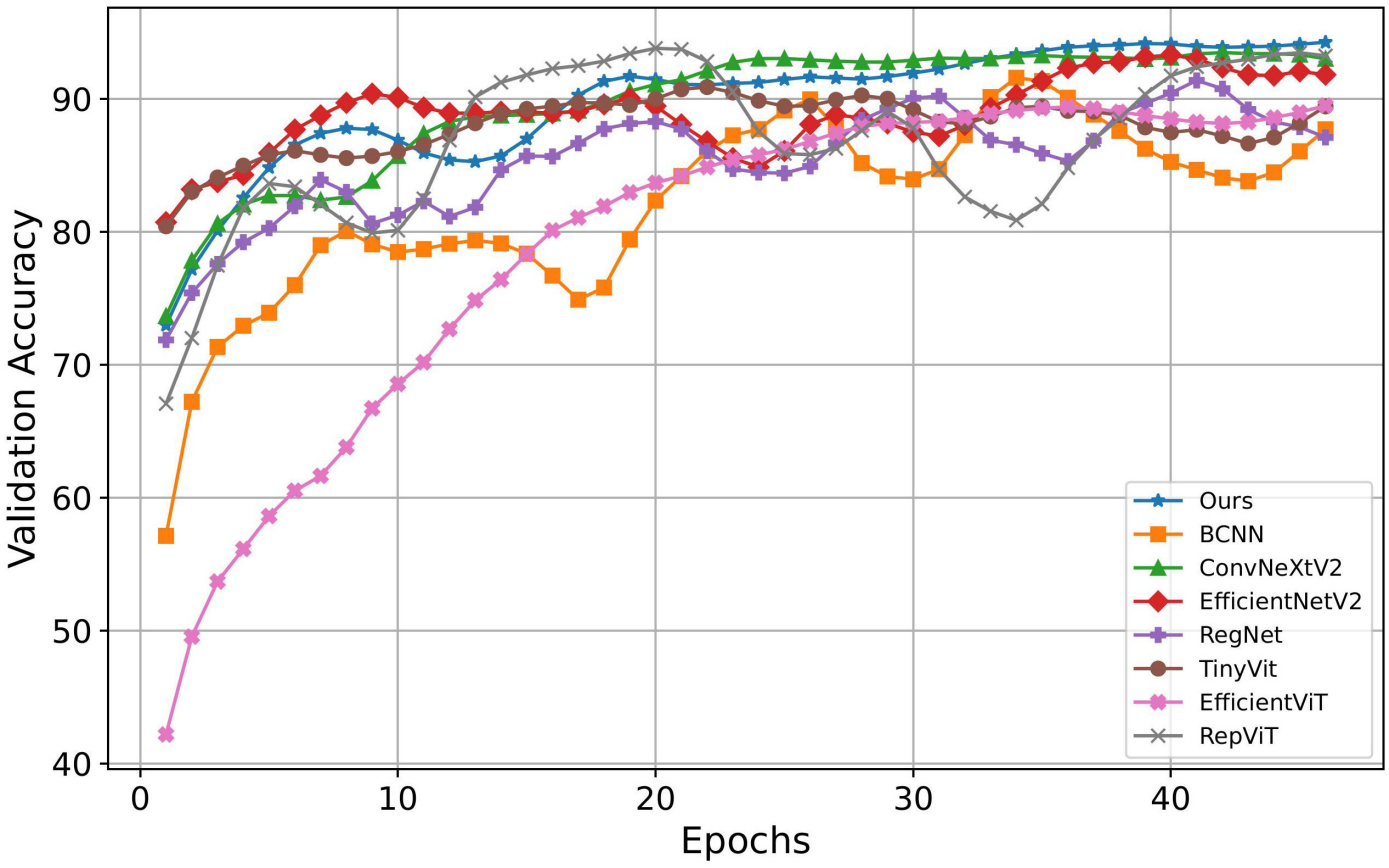
Convergence speeds of different methods.

On the other hand, this paper compared the iterative processes of different image classification models during training. The specific results are shown in **Figure 8**, where the horizontal axis represents the number of iterations, and the vertical axis represents the accuracy on the validation set. Based on the results in **Figure 8**, the proposed method demonstrates superior performance compared to other comparative methods. Although EfficientNetV2 and TinyVit converge quickly, their final accuracy falls below that of the proposed model. ConvNeXtV2’s curve closely resembles the proposed method’s curve but achieves slightly lower final accuracy. Although RepViT’s final results are similar to the model proposed, its convergence speed is slower, and it exhibits significant oscillations during training, affecting the model’s stability. In contrast, while EfficientViT shows a smoother upward curve and demonstrates a certain level of stability, its initial accuracy is relatively low, and its final accuracy is inferior to other models. These findings underscore the superior performance and training efficiency of the proposed method.

Additionally, this paper conducted experiments involving the merging of major Chrysanthemum categories and the subsets of Chrysanthemum morifolium ‘Hangbaiju’. The experimental results are illustrated in **Table 4**.

**Table 4:** Experimental results of different methods on the amalgamation of major Chrysanthemum categories and the subsets of Chrysanthemum morifolium ‘Hangbaiju’.

Method	Amalgamation of major Chrysanthemum categories				Subcategories of Chrysanthemum morifolium ‘Hangbaiju’			
	Acc	Recall	FI	STD	Acc	Recall	FI	STD
BCNN (2017)	92.2	87.3	87.6	10.1	90.8	90.5	90.4	4.32
RegNet (2020)	94.7	91.0	91.5	8.16	94.1	93.8	93.8	2.75
EfficientNetV2 (2021)	95.5	92.3	92.7	6.67	95.3	95.0	95.0	2.49
ConvNeXtV2 (2023)	94.3	89.8	90.4	8.79	93.8	93.1	93.2	4.07
TinyVit (2022)	95.2	91.7	92.4	7.22	94.1	93.9	93.7	2.67
EfficientViT (2023)	94.3	90.0	90.7	8.88	92.4	92.1	92.0	3.73
RepViT (2024)	95.7	93.0	93.4	7.27	93.8	93.6	93.6	3.07
Ours	95.8	93.3	93.5	5.79	95.9	95.3	95.7	2.60

As observed from **Table 4**, the proposed model demonstrates higher performance when dealing with the amalgamation of major Chrysanthemum categories. It achieved 95.8%, 93.3%, and 93.5% in accuracy, recall, and F1 score respectively, surpassing other models’ results. Additionally, the proposed model showcases good performance in terms of standard deviation, with a value of only 5.79. Although EfficientNetV2, TinyVit, EfficientViT, and RepViT achieve accuracy levels close to the proposed model, their recall or standard deviation (STD) show significant gaps compared to the proposed method. These results indicate that the proposed method exhibits better generalization and discrimination capabilities when major Chrysanthemum categories are amalgamated. The observations from **Table 4** indicate that within the subcategories of Chrysanthemum morifolium ‘Hangbaiju’, the proposed model performs exceptionally well, effectively distinguishing between these similar categories. While EfficientNetV2 shows performance metrics close to those of the proposed model, it even outperforms it in terms of standard deviation. However, ConvNeXtV2, TinyVit, EfficientViT, and RepViT do not match the performance of the proposed model. However, ConvNeXtV2 and TinyVit do not match the performance of the proposed model.

Analyzing these tables collectively, it’s clear that while EfficientNetV2 performs well with fewer categories, its performance diminishes with an increase in categories (as observed in **Table 3**). Conversely, ConvNeXtV2, RepViT, and TinyVit show overall accuracy rates similar to the proposed model with more categories, yet exhibit performance drops for specific or fewer categories. The proposed model demonstrates higher stability and reliability in classifying these diverse categories, effectively capturing intra-class feature differences. This superiority isn’t just evident in overall accuracy but also in discerning and capturing nuanced differences between categories, offering a more dependable and precise solution for Chrysanthemum classification.

### 4.4 Visual analysis

**Figure 9:**
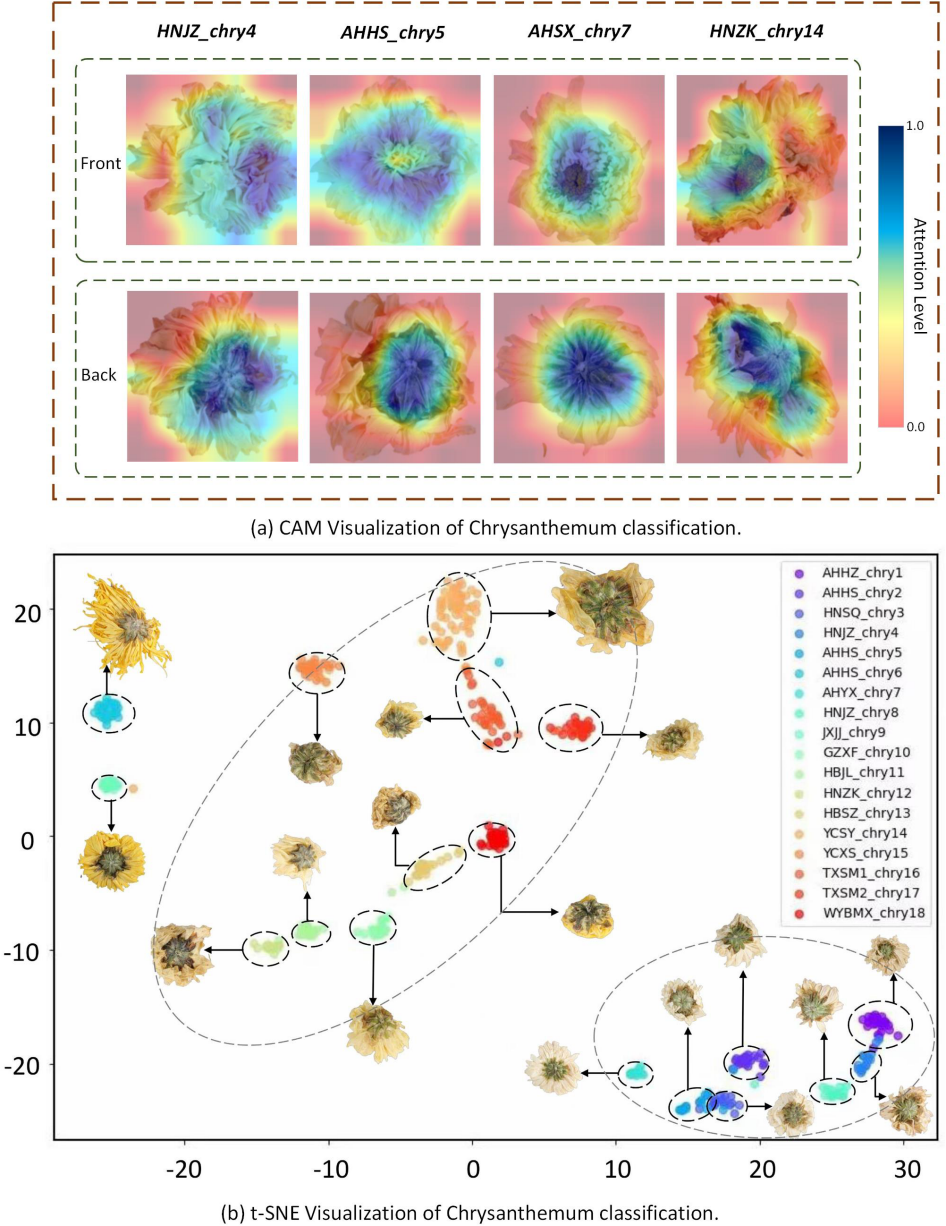
**(A)** Visualization of CAM. **(B)** Visualization of t-SNE.

This paper uses two visualization analysis methods to visualize chrysanthemum classification from two perspectives, as shown in **Figure 9**.

Class Activation Mapping (CAM) was used to generate heatmaps for the four types of chrysanthemum images, as shown in **Figure 9A**, where darker colors indicate areas of greater attention by the model. The heatmaps clearly show that, when processing the back view of the chrysanthemum, the model mainly focuses on the calyx, while for the front view, it primarily focuses on the pistil. This result strongly supports our earlier claim that the back view of the chrysanthemum contains rich detailed features, and that the front and back views offer complementary features. This complementarity further highlights the potential of fusing both views to enhance classification performance.

The paper utilizes t-SNE to generate a scatter plot illustrating the clustering of Chrysanthemum classifications, as shown in **Figure 9B**. The x-axis and y-axis represent specific features, with each point representing an individual Chrysanthemum sample. Different colored points denote distinct Chrysanthemum categories, displaying their distribution in the feature space. The visualization reveals two primary clusters within the Chrysanthemum dataset, accompanied by smaller clusters inside them, depicting similar shapes and corresponding to different Chrysanthemum categories. The first prominent cluster includes Hangbaiju like GZXF_chry10, HBJL_chry11, HBSZ_chry13, and YCSY_chry14. The second significant cluster includes Chrysanthemums like AHBZ_chry1, AHHS_chry2, HNSQ_chry3, and HNJZ_chry4. This visualization offers valuable insights into studying the differences among Chrysanthemum categories, facilitating model optimization for better category differentiation.

For instance, some small clusters are distinctly separated from others, such as AHHS_chry6 and JXJJ_chry9, indicating that the model demonstrates high accuracy in distinguishing these categories. Conversely, certain small clusters are very close to each other, particularly within the second-largest cluster, where the distances between AHHZ_chry1 and HNJZ_chry4, as well as HNSQ_chry3 and AHHS_chry5, are short. This suggests that the model experiences some confusion when distinguishing these categories, as reflected in the earlier confusion matrix showing misclassifications for these species. These observations provide clear directions for model optimization, allowing future work to focus on making specific adjustments for these hard-to-differentiate categories, thereby enhancing the overall performance and generalization of the model.

### 4.5 Stability analysis

**Figure 10:**
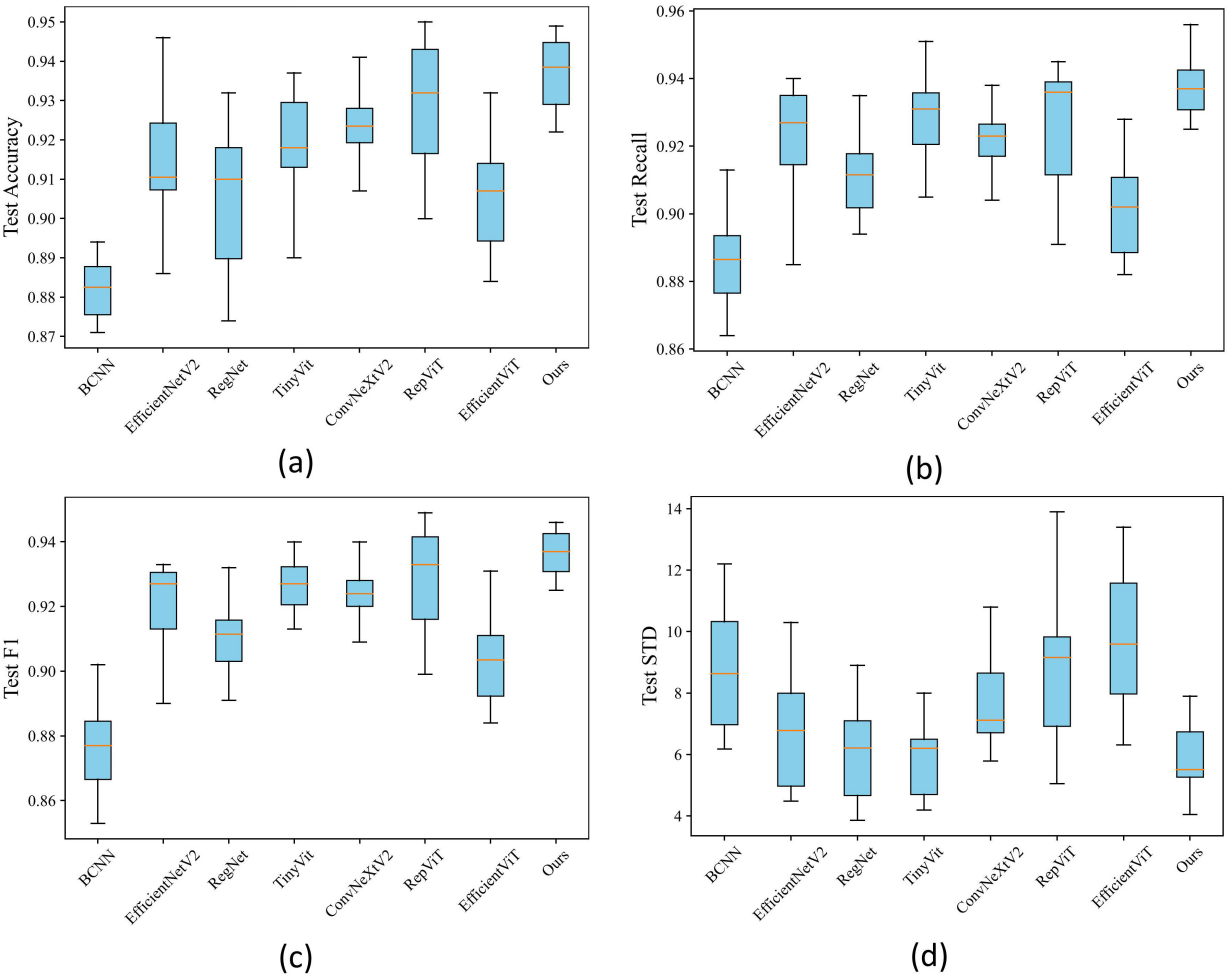
**(A)** Boxplot of accuracy. **(B)** Boxplot of F1 score. **(C)** Boxplot of recall. **(D)** Boxplot of standard deviation (STD).

To ascertain the stability of our proposed method, this paper created box plots comparing it with other methods in Chrysanthemum classification accuracy. As depicted in **Figure 10**, the horizontal axis of the box plot represents various image classification methods, and the vertical axis denotes the evaluation metrics corresponding to each method, illustrating the data distribution of the results. Based on the outcomes in A,B,C, EfficientNetV2, TinyVit, and RepViT attained suboptimal results, albeit with a gap in stability compared to the proposed method in this paper. However, while RegNet and ConvNeXtV2 exhibited higher stability, their performance across various indicators was inferior to the proposed method outlined in this paper. Figure D demonstrates that the proposed method excels in stability concerning inter-class variance, while RegNet and TinyVit lag slightly and ConvNeXtV2, RepViT, and EfficientViT diverges significantly from the proposed method. In summary, the proposed method demonstrates both high stability and accuracy, facilitating effective classification of Chrysanthemum images.

### 4.6 Ablation experiments

To assess the contribution of each component in the Chrysanthemum classification method proposed here, this paper conducted several ablation experiments. These included: 1) using front Chrysanthemum images only, 2) using back Chrysanthemum images only, 3) utilizing both front and back data simultaneously, 4) implementing cross-stream residual connections solely in convolutional layers, and 5) adjusting the weight ratio *α* of the two-stream summation in fully connected layers, with *α* values of 1, 1/2, 1/3, 1/4 (this paper used *α*=1/3 in this paper). These experiments aimed to analyze each component’s impact on method performance, offering a deeper insight into the method’s role and significance in Chrysanthemum classification. Detailed experiment results are outlined in **Table 5**.

**Table 5:** Experimental results of ablation experiments.

Method	Acc(%)	Recall(%)	F1(%)	STD
Front image only	85.5	85.0	85.0	11.7
Back image only	90.5	90.1	90.0	7.61
Both front and back image	91.0	90.0	90.1	8.47
Cross path residual connections	93.0	92.3	92.2	6.25
*α*=1	91.7	91.2	91.1	8.67
*α*=1/2	93.3	92.7	92.7	5.57
*α*=1/4	92.9	92.6	92.8	5.41
*α*=1/3(ours)	93.8	93.5	93.4	5.20

Through observing the results in the table, it can be concluded that solely using front-facing image features results in lower accuracy, reaching only 85.5%. However, employing back-facing image features leads to a significant performance boost, achieving an accuracy of 90.5%. This demonstrates an improvement in performance by using back-facing features alone but doesn’t fully exploit the model’s potential. Meanwhile, the performance slightly improves to 91.0% by using both front and back image features together, indicating that mere feature concatenation doesn’t fully accomplish feature fusion. In contrast, employing cross-stream residual connections in the convolutional layers better achieves feature fusion, elevating accuracy to 92.6%. Moreover, the weighted summation operation in the fully connected layers further enhances feature fusion, and we identified the most suitable weight ratio. The 1:3 weighted ratio exhibits the best performance, reaching the highest accuracy of 93.8%. These findings highlight the pivotal role of feature fusion in Chrysanthemum classification methods, offering significant insights into method performance. Overall, the comprehensive use of cross-stream residual connections and fully connected weighted summation effectively fuse image features from both sides of Chrysanthemum images. This approach accounts for the differences between front and back features among different Chrysanthemum types, achieving feature complementarity for more accurate Chrysanthemum classification.

## 5 Conclusion

This paper proposes a Chrysanthemum classification method based on the fusion of deep visual features of both the front and back sides. Different Chrysanthemum images are collected and labeled with origins and classifications. The front and back images underwent separate preprocessing and were used as inputs for a two-stream neural network. Leveraging single-stream residual connections and cross-stream residual connections expands the receptive field of the network and fully fuses the features of the front and back sides. Overall, the innovation of this paper lies in the introduced two-stream neural network model and the strategy of fusing deep features of the front and back sides of Chrysanthemums. The experimental results demonstrate the precision and robustness of this method. These findings provide strong support for its practical application in real-world agricultural and medicinal scenarios.

In future work, we plan to use distortion simulation techniques to construct a dataset that includes various visual distortions, simulating the complex conditions encountered in real-world scenarios. Training the model on this dataset will significantly enhance its robustness in handling challenging environments, such as noise or image degradation. To enhance the model’s generalization capabilities, we plan to incorporate meta-learning. This will enable the model to excel in chrysanthemum classification and quickly adapt to other medicinal plants, allowing for swift adjustments to new sample origins or species and improving its effectiveness in diverse classification tasks. For example, this method is particularly suitable for medicinal plants like Tangerine Peel Pericarpium Citri Reticulatae, which exhibit significant differences between the front and back features. By extracting color and texture features from both sides, the model can be used for origin tracing and quality assessment. These improvements will help the model better adapt to real-world applications, such as agricultural and medicinal plant classification.

## Data Availability

The datasets presented in this study can be found in online repositories. The names of the repository/repositories and accession number(s) can be found below: https://github.com/dart-into/CCMIFB.
