# Supplementary material for: Chrysanthemum classification method integrating deep visual features from both the front and back sides
Source: Front Plant Sci. 2025 Jan 21;15:1463113. doi: 10.3389/fpls.2024.1463113 (PMC11790631; doi:10.3389/fpls.2024.1463113)
Supplement: Supplementary file 1 [file DataSheet1.pdf]

Supplementary Material

1 SUPPLEMENTARY TABLES AND FIGURES

1.1 Tables

Table S1. The details of Chrysanthemum image dataset.

Abbreviation	Origin	Categories	Number
AHBZ_chry1	Anhui Bozhou	Boju	136
AHHS_chry2	Anhui Huangshan	Dabanju	104
HNJZ_chry3	Henan Shangqiu	Gongju	93
AHSX_chry4	Henan Jiaozuo	Huaiju	97
HNJZ_chry5	Anhui Huangshan	Gongju	87
AHHS_chry6	Anhui Huangshan	Jinsiju	124
JXJJ_chry7	Anhui Shexian	Qiyueju	116
YCXS_chry8	Henan Jiaozuo	Qibaiju	119
AHHS_chry9	Jiangxi Jiujiang	Taoju	113
HNSQ_chry10	Guizhou Xifeng	Hangbaiju	97
GZXF_chry11	Hebei Julu	Hangbaiju	112
HBJL_chry12	Henan Zhoukou	Hangbaiju	80
HNZK_chry13	Hubei Suizhou	Hangbaiju	96
HBSZ_chry14	Jiangsu Yancheng Sheyang	Hangbaiju	114
YCSY_chry15	Jiangsu Yancheng Xiangshui	Xiangju	217
TXSM1_chry16	Zhejiang Tongxiang Shimen1	Hangbaiju	115
TXSM2_chry17	Zhejiang Tongxiang Shimen2	Hangbaiju	105
WYBMX_chry18	Zhejiang Wuyi Baimuxiang	Hangbaiju	135
